# The Analysis of Particles at Low Accelerating Voltages (≤ *10 kV*) With Energy Dispersive X-Ray Spectroscopy (EDS)

**DOI:** 10.6028/jres.107.047

**Published:** 2002-12-01

**Authors:** J. A. Small

**Affiliations:** National Institute of Standards and Technology, Gaithersburg, MD 20899-8371

**Keywords:** electron probe analysis, low voltage analysis, particle analysis, scanning electron microscopy, x-ray microanalysis

## Abstract

In recent years, there have been a series of advancements in electron beam instruments and x-ray detectors which may make it possible to improve significantly the quality of results from the quantitative electron-probe analysis of individual particles. These advances include: (1) field-emission gun electron beam instruments such as scanning electron microscopes (FEG-SEMs) that have high brightness electron guns with excellent performance at low beam energies, *E*_0_ ≤ 10 keV and (2) high-resolution energy-dispersive x-ray spectrometers, like the microcalorimeter detector, that provide high-resolution (< 10 eV) parallel x-ray collection. These devices make it possible to separate low energy (< 4 keV) x-ray lines including the K lines of carbon, nitrogen and oxygen and the L and M lines for elements with atomic numbers in the range of 25 to 83. In light of these advances, this paper investigates the possibility of using accelerating voltages ≤ 10 kV, as a method to improve the accuracy of elemental analysis for micrometer-sized particles.

## 1. Introduction

In classical electron probe analysis schemes employing either a ZAF, bulk-sample *ϕ*(*ρz*), or Bence-Albee approach, both sample and standard are assumed to be infinitely thick with respect to the penetration of the electron beam and have flat polished surfaces. For such a semi-infinite plate, the corrections for the interaction of the electron beam with the sample and the subsequent x-ray emission can be calculated from simple geometric relationships. In the quantitative analysis of particles, the size and shape of the particle often cannot be controlled or accurately measured which results in two first-order particle effects that influence the generation and measurement of x rays from these samples. The first particle effect is the result of the finite size (mass) of the particle. The mass effect is related to the elastic scattering of the electrons and is strongly affected by the average atomic number of the particle. The mass effect is important when the particle size is smaller than the range of the primary electron beam so that a significant fraction of the beam escapes the particle before exciting x rays. The second particle effect is the result of x-ray absorption, which is dependant on the shape of the analyzed particle. In the analysis of most particles, the x-ray emergence angle and therefore the absorption path length cannot be determined as it can for polished specimens. The magnitude of this effect is largest when there is high absorption as is typically the case for “soft” x rays from elements like Al or Si that have energies less than about 2 keV [1,2]. (A third particle effect due to secondary fluorescence is considered at most a second order effect and is not covered in this paper.)

**Fig. 1:**
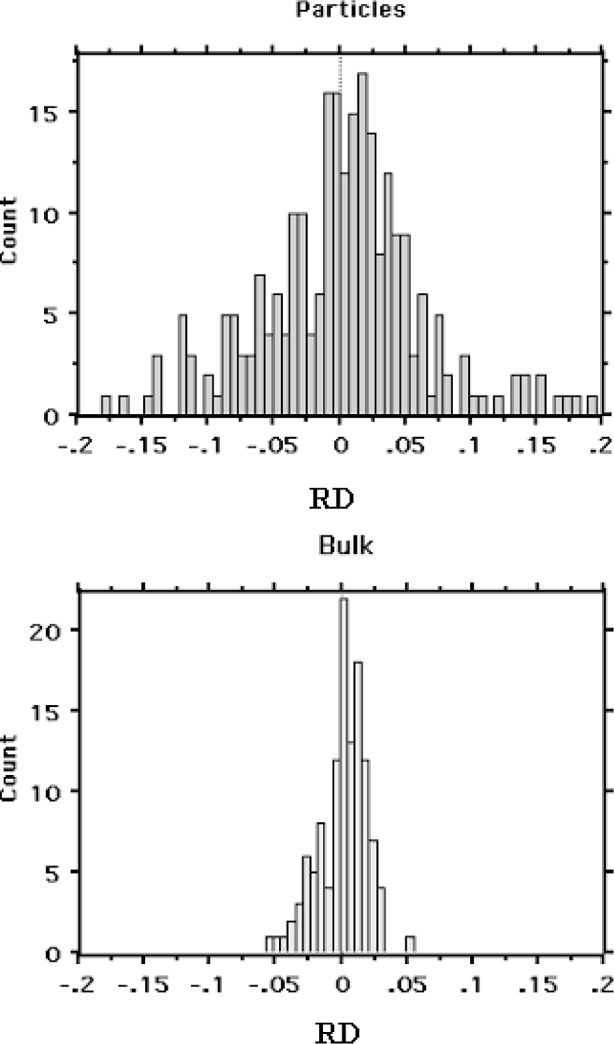
Relative error distributions for the analysis of glass particles and bulk glass samples.

Over the years, several researchers have developed correction procedures to minimize the particle effects and reduce the uncertainties associated with particle analysis. These procedures range from simple normalization of the elemental weight fractions obtained with conventional bulk-sample analysis procedures [3] to elaborate correction procedures based on modeling particle size and shape [4]. Although these correction procedures significantly reduce the effects of particle geometry, elemental concentrations determined from the quantitative analysis of particles by EDS are often characterized by relative uncertainties on the order of ± 0.10 to ± 0.20. This compares to relative uncertainties of ± 0.02 to ± 0.05 for the EDS analysis of bulk polished samples as shown in Fig. 1 for the analysis of glass particles and bulk glass samples where RD refers to the relative differences between the experimentally determined concentrations and the known concentrations (see Sec. 1.3.1) [5].

### 1.1 Particle Analysis at Lower Electron Beam Energies

In conventional electron probe microanalysis the accelerating voltage is generally in the range of 15 kV to 25 kV, which provides the necessary overvoltage and sufficient current from the thermionic source to excite efficiently the K and L x-ray lines for elements with atomic numbers as high as *Z* = 83. In general, this same criterion has been applied to the analysis of particles and the development of the methods used to analyze them.

The FEG-SEM in conjunction with a high resolution energy-dispersive x-ray detector such as the microcalorimeter detector will make it possible to use accelerating voltages in the range of 1 kV to 10 kV depending on the specific elements to be measured, to excite lower-energy characteristic x-ray lines with energies less than about 4 keV. Employing the L and M x-ray lines (energies from 0.640 keV to 2.42 keV) for the elements with atomic numbers in the range 25 to 83, rather than the K and L x-ray lines of these elements (energies 5.85 keV to 10.83 keV) should reduce significantly the magnitude of both the particle mass and absorption corrections.

**Fig. 2:**
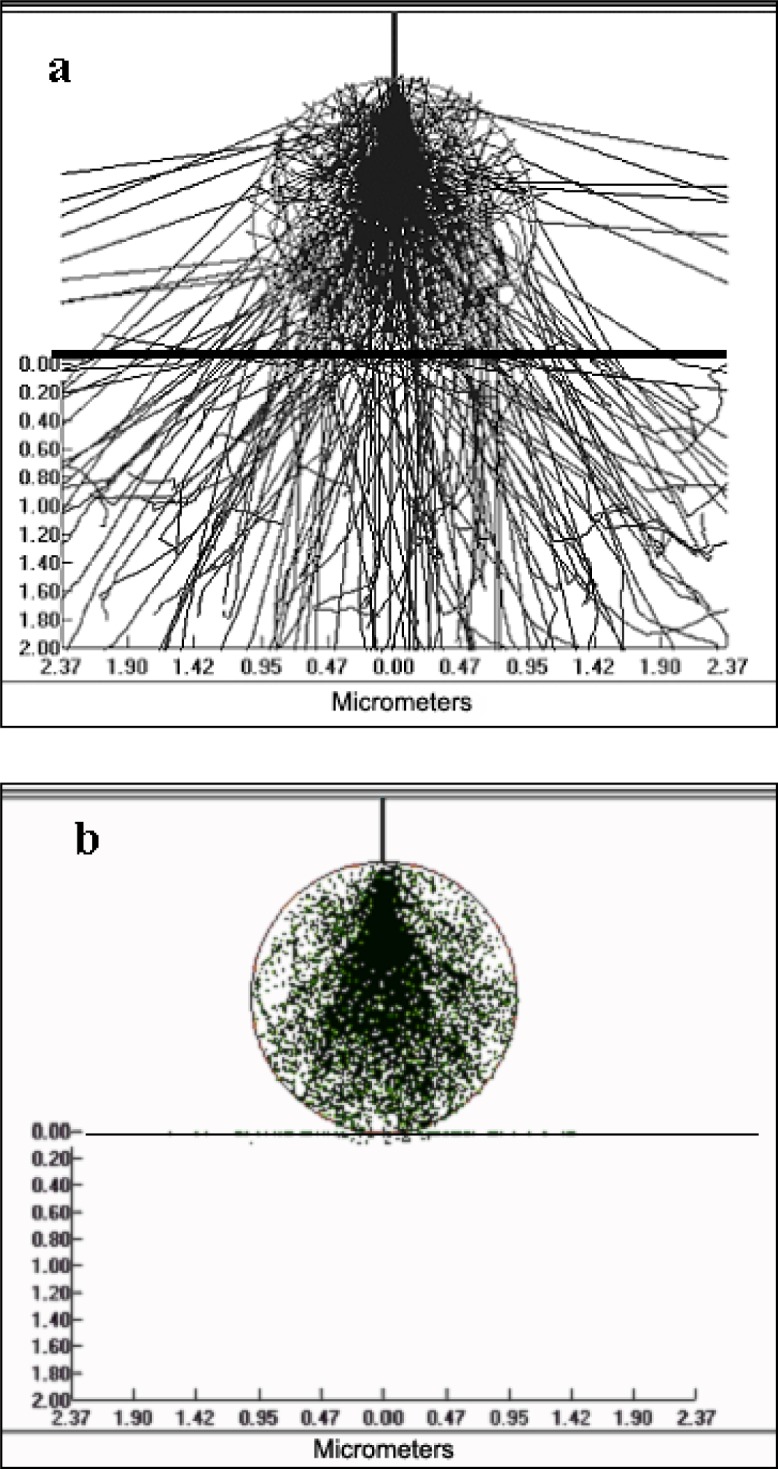
Monte Carlo plots for the interaction of a 20 kV electron beam with a 2 μm K-411 particle. a) Electrons trajectories. b) Mg Kα x-ray generation.

**Fig. 3:**
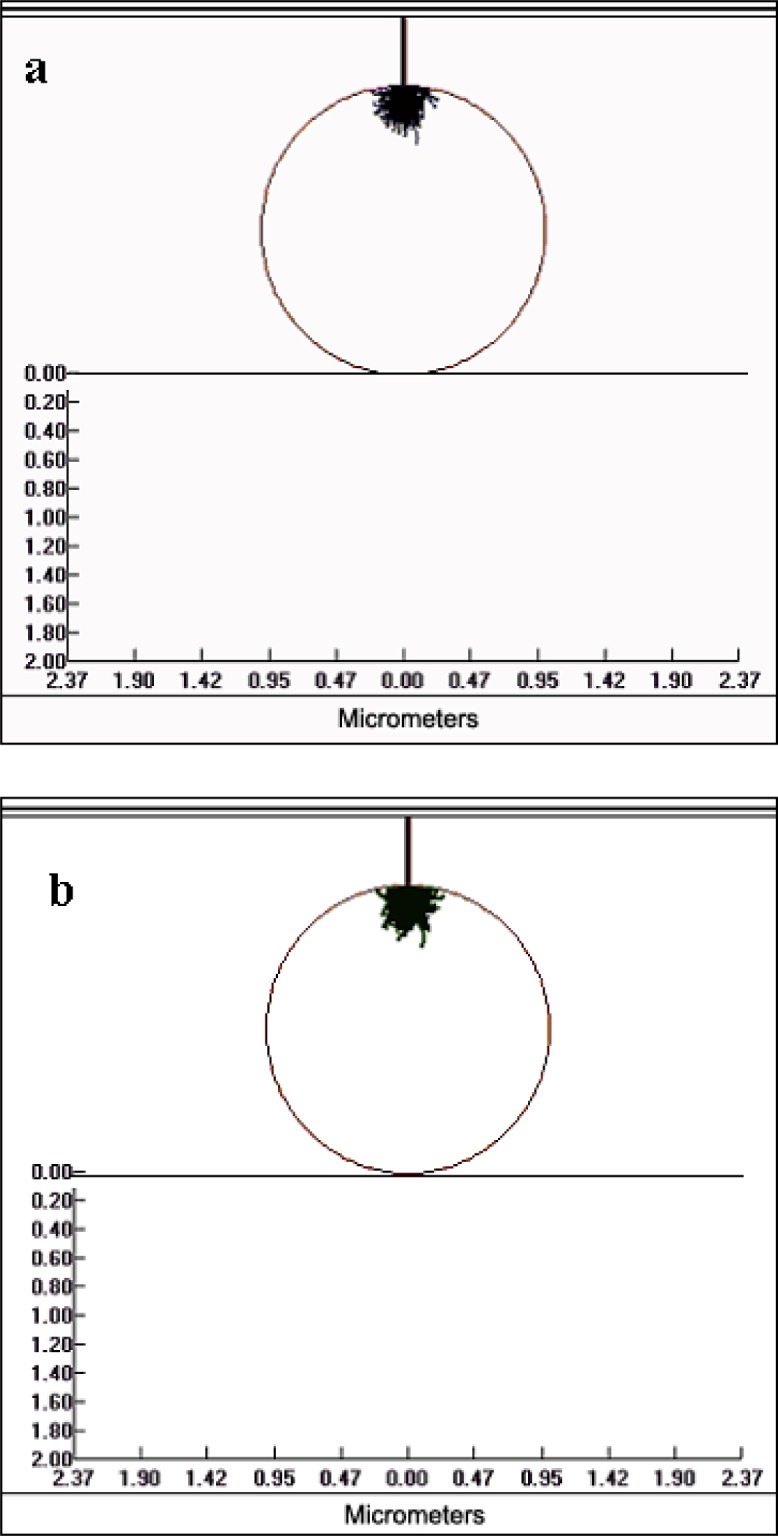
Monte Carlo plots for the interaction of a 5 kV electron beam with a 2 μm K-411 particle. a) Electrons trajectories. b) Mg Kα x-ray generation.

The advantage of reducing the electron beam energy when analyzing particles is that the electron beam interaction volume within the particle is reduced and therefore the x-ray generation volume for the particle will be similar to that from a bulk material, despite the particle geometry. This is illustrated in Figs. 2 and 3 which are Monte Carlo plots from the Electron Flight Simulator program version 3.1E (Small World Inc.^1^) based on the Monte Carlo model of Joy [6]. Figure 2 shows the electron trajectories (Fig. 2a) and the Mg Kα x-ray generation locations (Fig. 2b) for a 20 kV analysis of a 3 μm K-411 glass microspheres [7]. At 20 kV, the electrons interact throughout most of the particle volume with a significant fraction scattering out of the sides and bottom of the particle. In addition, a significant number of x rays are generated along the sides of the particle. As a result, the characteristic x-ray intensities emitted from this particle will be influenced by the particle’s shape and size, and will require corrections for both particle mass and particle absorption effects. The plots in Fig. 3(a) and 3(b) show that the electron interaction and Mg Kα x-ray generation volumes at 5 kV are considerably smaller than the volumes in the 20 kV plots. In fact they are small enough that the effects of particle shape and size are minimal and the particle analyzed under these conditions closely approximates a bulk material.

### 1.2 Experimental

To study the effects of reducing the electron beam energy for particle analysis, three different morphological forms of the analytical glass K-411, NBS SRM 470, which has the composition listed in Table 1, were analyzed [8].

**Fig. 4:**
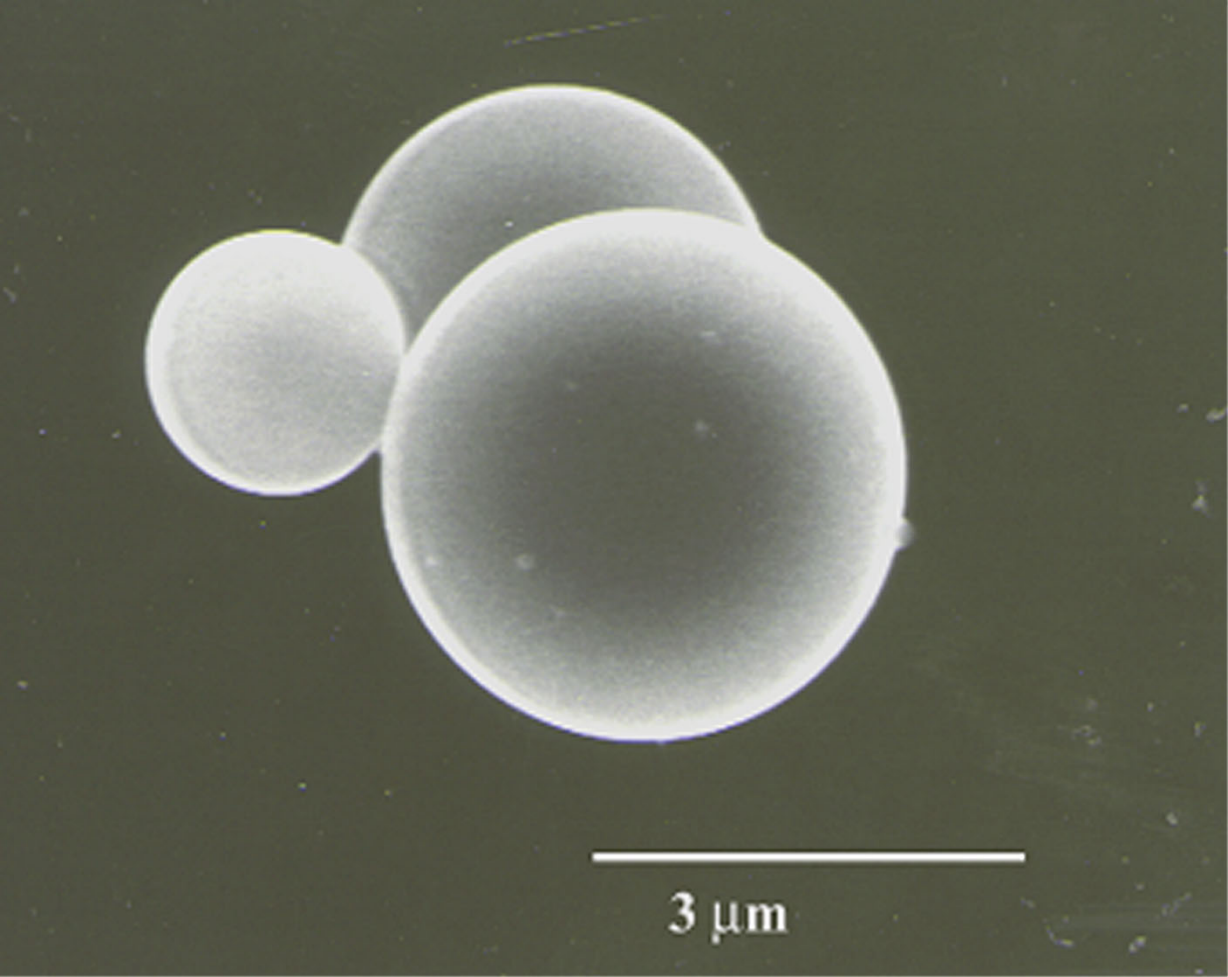
Secondary electron image of K-411 microspheres.

The morphological forms included bulk-polished glass, microspheres, and randomly shaped shards. The shards were prepared by dry grinding glass K-411 in a tungsten-carbide mortar and the microspheres were prepared by injecting the glass shards into a tube furnace [8,9]. Figure 4 is a secondary electron image of representative microspheres, which range in size from < 1 m to > 100 μm in diameter. The shards and the microspheres were dispersed onto conductive carbon tape; the bulk glass was mounted in Ag epoxy and polished. All samples were coated with approximately 10 nm of carbon to reduce charging effects.

The bulk glass and the microspheres were analyzed in an Hitachi S-4500 FEG-SEM equipped with a PGT IMIX system including a 60 mm^2^ Prism x-ray detector. Analyses were done at accelerating voltages of 5 kV and 10 kV, for 500 s and at 15 kV, and 25 kV for 300 s. The deadtime during spectrum collection was generally less than 20 %. Microspheres 1 μm to 30 μm in diameter were analyzed using an area raster and the results reported as the ratios of the background-corrected, elemental x-ray peak intensities from the microspheres divided by the corresponding bulk-glass intensities, (particle/bulk) ratios. Background correction was performed with a sequential simplex fitting procedure [10]. All measurements were corrected for drift in beam current. Different microspheres were analyzed for each voltage in an attempt to minimize contamination from the beam raster.

Four irregularly shaped K-411 glass shards ranging in size from about 1 μm to 24 μm were analyzed in spot mode for 300 s at 10 keV, 17 keV, 20 keV, 22 keV, and 25 keV in a JEOL 8600 electron probe equipped with a Tracor Northern x-ray detector. Quantitative electron probe analysis was run on the shards employing the ZAF quantitative correction procedure contained in NIST-NIH Desk Top Spectrum Analyzer (DTSA) [10]. Standards consisted of bulk, polished materials and included MgO for Mg, SiO_2_ for Si, K-412 glass, SRM 470, [8] for Ca, and K-411 glass for Fe. An MLLSQ-fitting procedure was used with reference peak shapes for background subtraction [10].

In addition to the K-411 shards, shards of K-3189 glass (Table 2) were also analyzed in the JEOL 8600 electron microprobe. The K-3189 particles were analyzed for 500 s at 10 kV and 20 kV utilizing both an overscan of the particle area and a spot analysis on the particle center.

### 1.3 Results and Discussion

#### 1.3.1 Particle Mass Effect

**Fig. 5:**
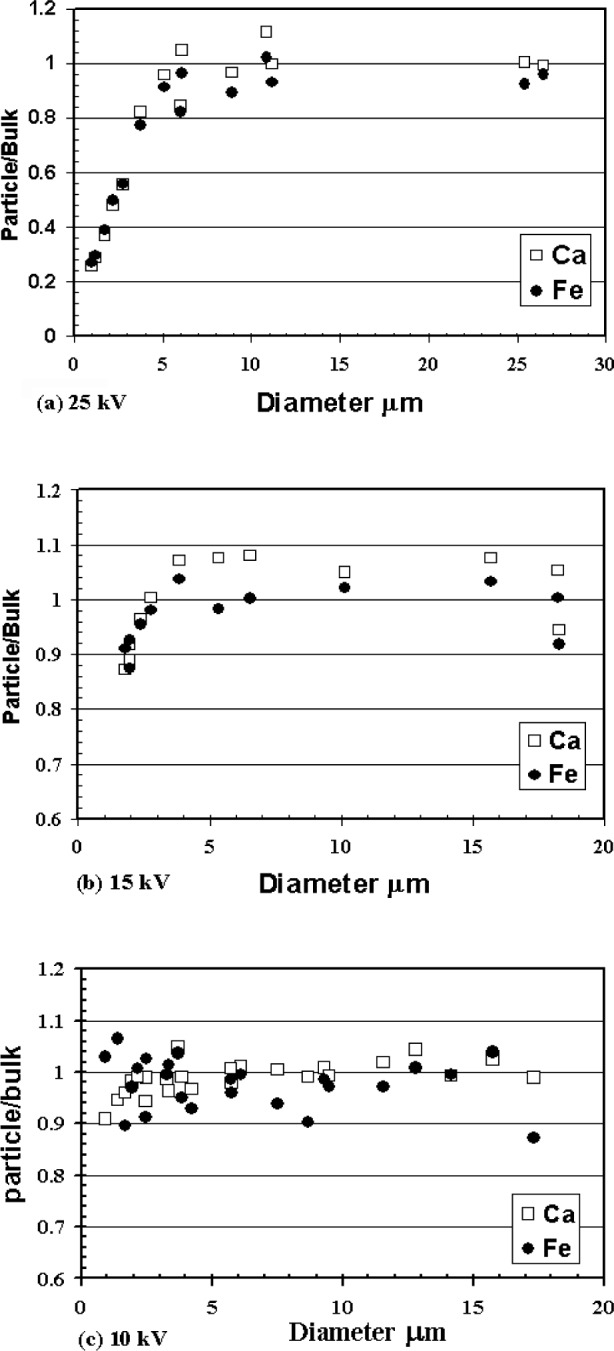
Plots of Fe and Ca Particle/Bulk values vs particle diameter for K-411 microspheres at different kV. (a) 25 kV, (b) 15 kV, (c) 10 kV.

The effect of reducing the acceleration voltage on the magnitude of the particle mass correction can be seen in Fig. 5 which shows the particle/bulk ratios for the Kα x-ray lines of Ca (3.47 keV) and Fe (6.40 keV) vs particle diameter at the different beam energies. These x-ray lines are energetic enough that the particle absorption correction is minimal and the shapes of the various plots are dominated by the particle mass correction. The 25 kV plot, Fig. 5(a), shows that the Fe and Ca curves approach unity only for particles with diameters greater than about 5 μm. Below this diameter, the particle mass effect dominates due to electron scatter out the bottom and sides of the particles. This results in a 50 % loss in x-ray intensity for a 2 μm sphere and a 75 % loss for the smallest microspheres ≈1 μm in diameter.

At 15 kV, Fig. 5(b), the magnitude of the particle mass effect is significantly reduced. At this voltage, the maximum loss of x-ray intensity is only about 13 % for a 1.8 μm diameter sphere compared to the 50 % drop for a 2 μm sphere observed in the 25 kV data. In addition, the particle/bulk curves for the 15 kV data approach unity (values within 0.9 and 1.1) for the particles greater than about 3 μm in diameter compared to 5μm observed at 25 kV.

Lowering the accelerating voltage to 10 kV further reduces the magnitude of the particle mass correction as shown in Fig. 5(c). The maximum loss of x-ray intensity for the 10 kV data is only about 10 % for all of the particles (excluding the one anomalous Fe value for the 18 μm sphere which is at 0.87). Unlike the 15 kV and 25 kV plots, the Fe and Ca particle/bulk values at 10 kV remain near unity for all of the particles in the size range analyzed, 0.9 μm to 18 μm with no obvious drop off even for the smaller diameter particles.

#### 1.3.2 Particle Absorption Effect

**Fig. 6:**
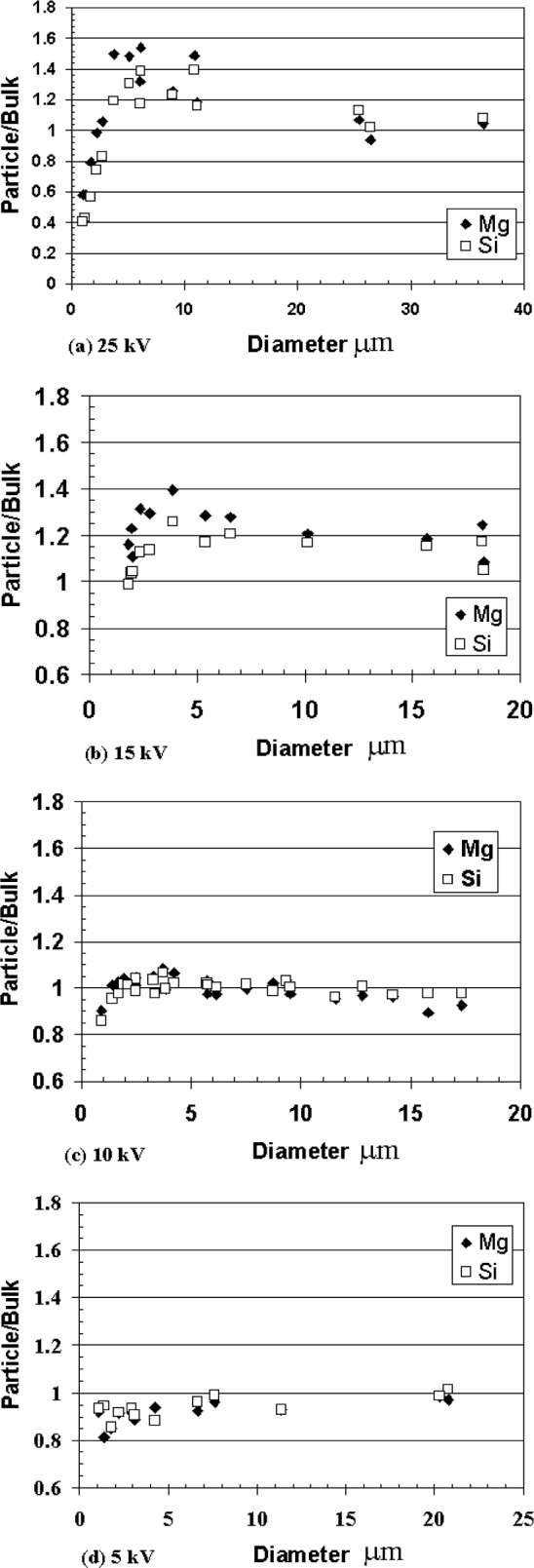
Plots of Mg and Si Particle/Bulk values vs particle diameter for K-411 microspheres at different kV. (a) 25 kV, (b) 15 kV, (c) 10 kV, (d) 5 kV.

The effect of reducing the acceleration voltage on the magnitude of the particle absorption effect can be seen in Fig. 6, which plots the particle/bulk ratios as a function of particle size for the Kα x-ray lines of Si (1.74 keV) and Mg (1.24 keV). The energies of these x-ray lines are low enough that x-ray absorption is significant and the particle absorption effect dominates for microspheres larger than about 4 μm in diameter. For particles less than about 4 μm the particle mass effect would still dominate for the higher beam energies.

At 25 kV, Fig. 6(a), the Mg particle/bulk values are > 1 for all particles larger than 3 μm reaching a maximum of about 1.6 for a 5 μm sphere. This ratio decreases to approach 1 only for the largest spheres with diameters ≥25 μm. The Si particle/bulk values behave similarly to the Mg values reaching a maximum of 1.4 for a 6 μm sphere, and decreasing to near 1 for the larger spheres. As expected, the Si particle/bulk ratios have a lower maximum compared to the Mg values since the Si x rays are slightly more energetic than the Mg x rays resulting in a smaller absorption effect. As mentioned the particle mass effect dominates for particles less than about 4 μm in diameter, as evidenced in both the Mg and Si plots with the particle/bulk values < 1 for the smaller particles.

The 15 kV results, Fig. 6b, indicate a significant reduction in the particle absorption effect compared to 25 kV results. The maximum particle/bulk value for Mg at 15 kV is only about 1.25 for a 4 μm sphere compared to the value of 1.5 for the 5 μm sphere at 25 kV. In addition, the Mg particle/bulk values now approach 1 for spheres in the 10 μm size range with particle/bulk values of 1.1 for a 10.5 μm sphere and 1.05 for a 15.5 μm sphere. The Si data at 15 kV has a maximum particle/bulk value of 1.18 for a 4 μm sphere compared to the 1.4 for roughly the same size sphere at 25 kV. In addition, the Si particle/bulk ratios for the two large spheres, 10.5 μm and 15.5 μm, are equal to 1 within the experimental uncertainties.

Similar to the particle mass effect, the particle absorption effect is even further reduced at 10 kV, Fig. 6(c). With the exception of a drop in the particle/bulk ratio for the smallest particle at 0.93 μm, due to the particle mass effect, the particle/bulk curves for both Si and Mg at 10 kV are relatively flat for all particle sizes compared to the 15 kV and 25 kV plots. In addition, the particle/bulk values for the 10 kV plot do not show any significant trend above 1 for particles in the 2 μm to 4 μm size range as was evident in the 15 kV and 25 kV plots. For the 10 kV data, the particle/bulk values for Si and Mg are all between 0.9 and 1.1 for all particles greater than 1.4 μm.

**Fig. 7:**
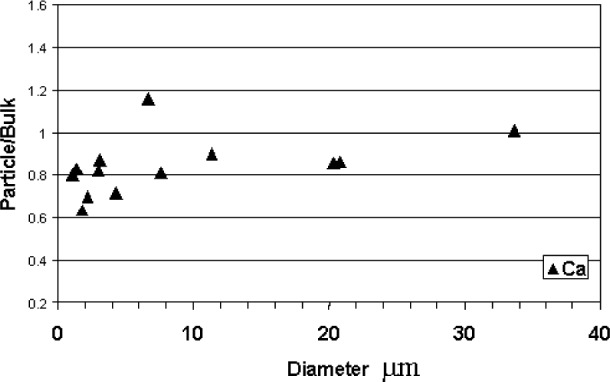
Ca Particle/Bulk ratios at 5 kV.

The results for 5 kV, Fig. 6d, are similar to the 10 kV data with relatively flat curves. However it is not possible to determine if there is any significant improvement compared to the 10 kV data since the Mg and Si particle/bulk values show an unexpected and significant bias < 1 for the smaller particles. This behavior is also evident in the Ca Kα particle/bulk ratios, shown in Fig. 7, for which x-ray absorption is not an issue. This anomaly, which is not evident in the 10 kV plots for these elements, is believed to be the result of sample contamination rather than the result of particle mass or absorption effects and is explained in greater detail in the section on contamination later in this paper.

Attempts to use the Fe L line at 5 kV for tracking the absorption effect were not successful because the Fe L intensities were too low resulting in high variations in the net (background corrected) peak intensities even for bulk glass analyses.

#### 1.3.3 Quantitative Analysis of Glass Shards

In addition to the measurement of particle/bulk ratios for the glass microspheres, four K-411 glass shards were quantitatively analyzed in the electron probe. Quantitative analysis was performed with a conventional bulk-sample correction algorithm that involved the comparison of the x-ray intensities from the particles to the intensities from bulk standards. The relative difference (*RD*) between the particle values and the certified bulk values for all elements were then calculated from the following equation:

RD=[(experimental−known)/known]. (1)

**Fig. 8:**
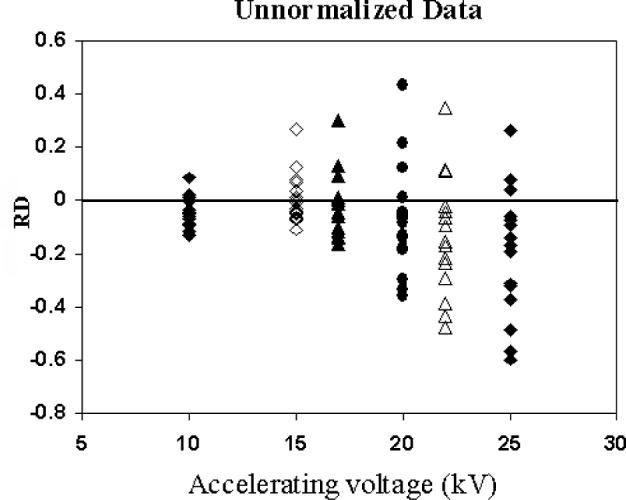
RD values for glass shards vs electron beam accelerating voltage.

The shards were analyzed at five different accelerating voltages ranging from 10 kV to 25 kV. The results of the analysis are shown in Fig. 8, which is a plot of the *RD*s observed in the quantitative results for all elements at each of the different beam energies. The *RD* values in Fig. 8 are for quantitative results without any normalization or corrections for particle effects. At 25 kV, the standard deviation of the *RD* values is 0.238 with *RD* values ranging from + 0.26 to − 0.60. The variations of the *RD* values remain large for 22 kV and 20 kV with standard deviations of 0.216 and 0.203, respectively. In contrast, the standard deviations of *RD* distributions for beam energies less than 20 kV drop dramatically. The standard deviations for the *RD* distributions at 17 kV and 15 kV are 0.120 and 0.094, respectively. At 10 kV the range of *RD* values is only from + 0.08 to − 0.13 with a standard deviation of 0.054.

**Fig. 9:**
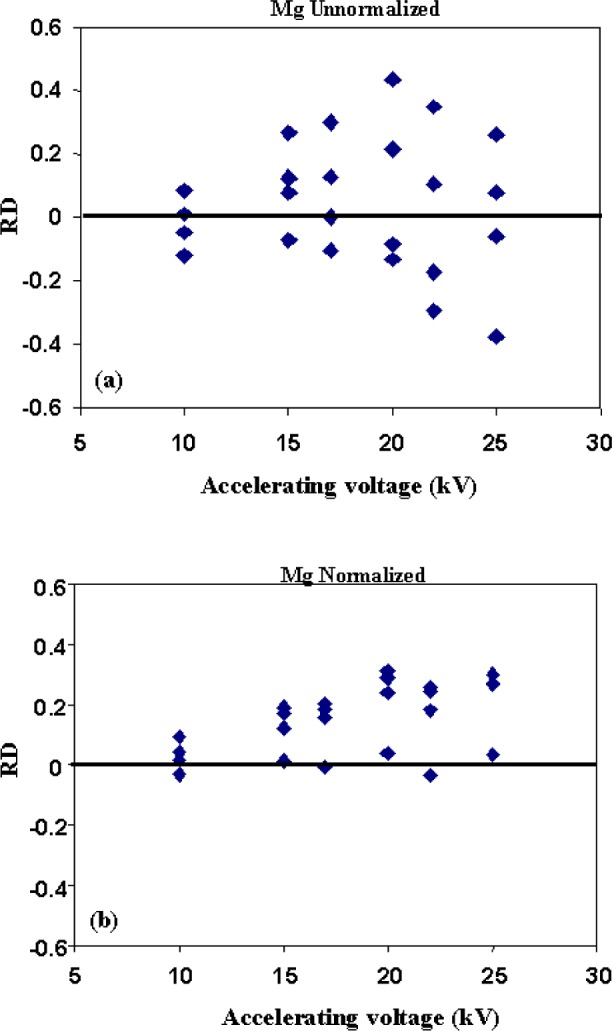
RD values for (a) unnormalized and (b) normalized Mg analyses vs accelerating voltage.

**Fig. 10:**
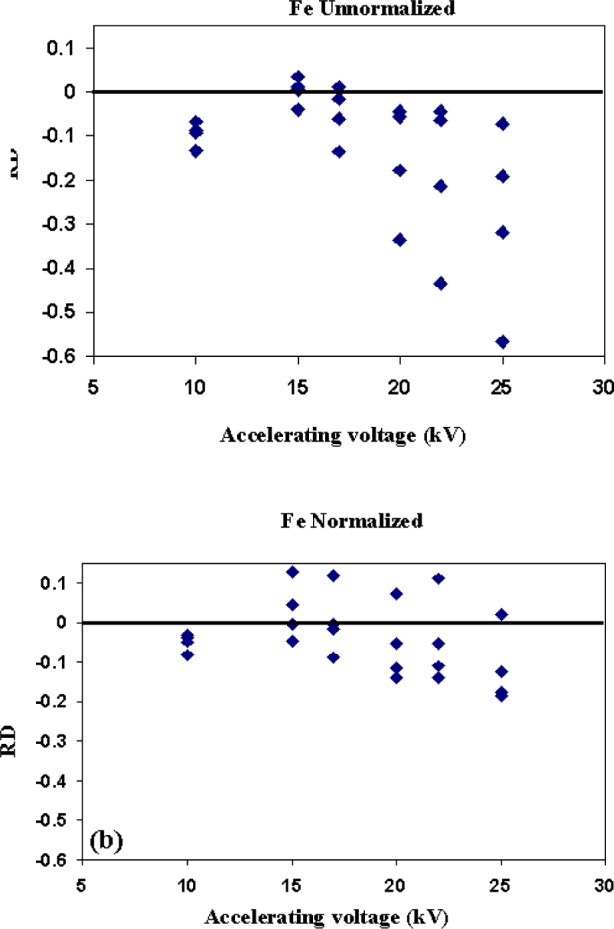
RD values for (a) unnormalized and (b) normalized Fe analyses vs accelerating voltage.

In addition to the pooled results for all the elements, the *RD* values for Mg, the lowest energy x-ray line for this glass composition, and Fe the highest energy line are shown in Figs. 9 and 10. The *RD* values for the unnormalized Mg analyses on the four shards are plotted in Fig. 9(a) as a function of beam energy. The variations of *RD* values for 22 kV and 25 kV are the largest with ranges of 0.63 and 0.64, respectively. As expected, the smallest range is 0.20 for 10 kV since the lower acceleration potential minimizes the particle effects.

Figure 9(b) shows a plot of the *RD* values for Mg analyses in which the sum of the various elemental concentrations have been normalized to 100 %. For these calculations oxygen was determined by stoichiometry. Normalizing the particle data reduces the range of the *RD* values, for all voltages. For example, at 25 kV the distribution of *RD* values ranges from 0.63 (−0.37 to + 0.26), to 0.34 (+ 0.036 to + 0.30) and for 10 kV the *RD* values only range from 0.20 (−0.12 to + 0.08) to 0.13 (−0.035 to + 0.095). However, while the range of the results improves with normalization, the normalized results show a systematic positive offset with the majority of the *RD* values for the different voltages being > 1. The positive offset is due to the particle absorption effect and is expected since simple normalization corrects mainly for the particle mass effect, i.e., the negative *RD* values in the unnormalized data, but not for absorption (Small 1981).

The unnormalized Fe data are shown in Fig. 10(a). For the Fe data the magnitude of the *RD* distributions follows a similar trend to the Mg data with the largest variation in *RD* values exhibited for the 25 kV data at 0.50 and the smallest variation for the 10 kV data at 0.065. Unlike the Mg data, the *RD* values in the unnormalized Fe values are all negative except the 15 kV data which are distributed around zero. The observed systematic negative *RD* values are the result of the particle mass effect but only for the voltages 17 kV and above. The anomalous negative displacement for the 10 kV Fe data is believed to be the result of contamination similar to the anomalous results shown in Figs. 5d and 6 for the 5 keV data on Mg, Si, and Ca and not related to the particle mass or absorption effects (see the discussion of contamination below).

Looking at the normalized data, Fig. 10(b), the range of *RD* values is smaller than the unnormalized data with ranges in the *RD* values of 0.21 for 25 kV to only 0.05 for the 10 kV data. Since the dominant effect on the Fe data is the mass effect, the normalization reduces the spread in *RD* values and also significantly reduces the systematic negative offset in the unnormalized Fe *RD* distributions for the higher beam energies. It is important to note that for the 10 kV data the normalization does not correct the negative offset in the data. This further indicates that the observed trend is not related to the particle mass effect.

#### 1.3.4 Surface Contamination Effects in Low-Voltage Analysis

The use of lower accelerating voltages for particle analysis can significantly reduce the magnitude of the particle mass and absorption effects and improve the quantitative results as shown above. However, reducing the accelerating voltage does introduce systematic errors in the measured x-ray intensities, unrelated to particle effects, which must be taken into account to obtain the highest accuracy in quantitative analysis. The systematic errors are the result of the formation of a contamination layer on the particle surfaces during analysis with the electron beam. This problem, although not unique to particles, can have a pronounced effect when analyzing micrometer-sized or smaller particles because the electron beam is often focused to a spot or rastered over a very small area for relatively long counting times often in excess of 500 s for the smaller particles. In this case, comparing the particle x-ray intensities to bulk-standard intensities, where the beam is often rastered over a much larger area and for a shorter time, results in systematic negative bias in the *k*-ratios. The primary effect of the contamination layer is not an increased absorption of generated x-rays but a retardation or reduction of the average energy of the electron beam that actually enters the specimen and hence the reduction of the overvoltages for the various analytical lines. The overvoltage is defined as: *U* = *E*_0_/*E*_crit_, where *E*_0_ is the energy of the electron beam and *E*_crit_ is the critical excitation energy of the x-ray line. The x-ray intensity is observed to follow a general form, *I* ∝ (*U* − 1)^n^, where *n* ~ 1.5.

The recommended minimum overvoltage is 2 V for conventional quantitative electron probe analysis with values as low as 1.5 V in special circumstances [11]. As the acceleration voltage is lowered to minimize the particle effects, the overvoltages for the various characteristic x-ray lines also decrease. In many cases, this necessitates choosing a characteristic line for which the overvoltage is less than the recommended value. The lower the overvoltage the greater will be the effect of electron retardation resulting in lower than expected characteristic x-ray intensities for all elements.

**Fig. 11:**
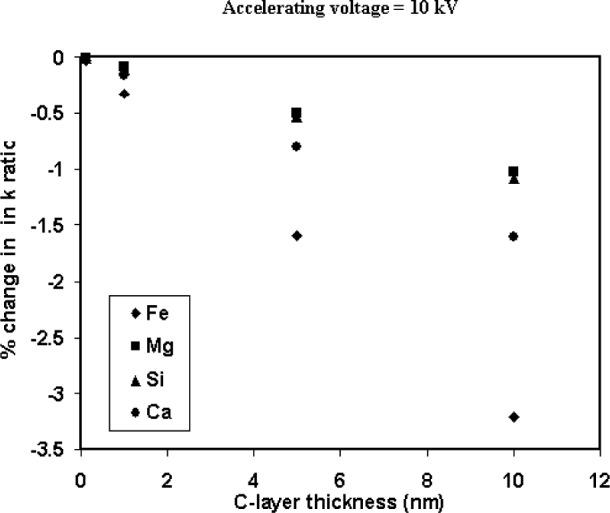
Monte Carlo calculations of the percent change in k-ratios for elements in K-411 glass as a function of carbon thickness.

In this study, the anomalies observed in the quantitative analyses for Fe in the shards (Fig. 10), and Ca particle/bulk plot at 5 kV (Fig. 7) are thought to be manifestations of this contamination effect. A beam energy of 5 keV is reasonably low but for Mg and Si the overvoltages are greater than 2.7 V. In this instance, the effect of contamination is relatively modest with particle/bulk ratios on the order of 5 % low for the microspheres < 5 μm as seen in Fig. 6(d). Monte Carlo calculations [12] of the elemental *k*-ratios for K-411 glass coated with a varying thickness of carbon (Fig. 11) indicates that there is a 2 % drop in the Mg and Si *k*-ratios from a 5 nm thick layer of C increasing to roughly 5 % for a 10 nm thick layer.

The systematic offset in the Fe data from the shards analyzed at 10 keV, Fig. 10(a), is on the order of −10 % relative. Since 10 kV is not a particularly low-accelerating voltage the effect of a contamination layer on the average beam voltage is minimal. However, the overvolatge for Fe at 10 kV is only 1.5 V, at the lower end of the recommended values for conventional probe analysis, so that even a relatively small reduction in the average beam voltage results in a noticeable loss of Fe intensity from the particles. The contamination effect is evident only for the data collected on the electron probe and not for the analyses performed in the FEG-SEM. This was attributed in part to the formation of a thicker contamination layer in the probe due to the poorer chamber vacuum, which is on the order of 6 × 10^−4^ Pa in the probe compared to 6 × 10^−5^ Pa in the FEG-SEM. In addition a second possible source of the sample contamination may be the deposition, under beam bombardment, of hydrocarbon residue from particle mounting procedures as well as from the sample itself. The *k*-ratios for the Fe Kα x rays at 10 keV (Fig. 11) indicate that the Fe *k*-ratio has the most significant drop of all the elements with a 1.6 % decrease for a 5 nm C layer increasing to 3 % for a 10 nm layer.

To test further the effects of sample contamination on the analysis for elements such as Fe with low overvoltages, shards of glass K-3189 were analyzed at 10 kV and 20 kV using both an overscan of the particle area and a spot analysis on the particle center. The results are reported as the mean and standard deviation for Fe/Ca and Fe/Si intensity ratios in Table 3 and plotted in Figs. 13 and 14. The Fe intensities were normalized to the intensities for Si and Ca which have overvoltages that range from 5.44 for Si to 2.48 for Ca both well above the 1.5 limit so that their peak intensities will not be significantly affected by a reduction in the electron beam energy due to sample contamination.

**Fig. 12:**
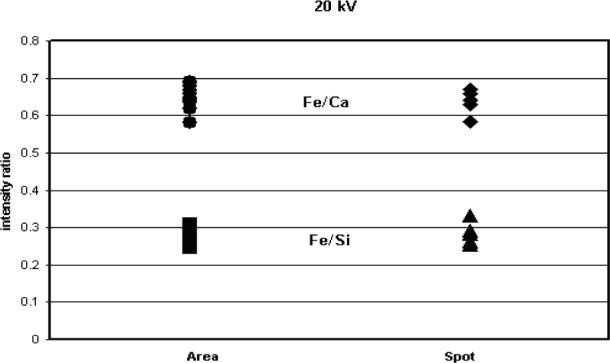
Plot of Fe/Ca and Fe/Si intensity ratios for the area and spot analyses of glass K-3189 at 20 kV.

**Fig. 13:**
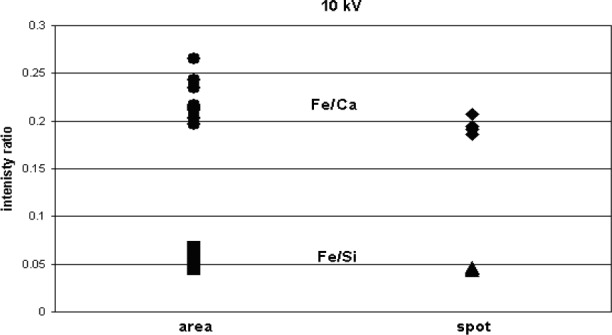
Plot of Fe/Ca and Fe/Si intensity ratios for the area and spot analyses of glass K-3189 at 10 kV.

The results from the K-3189 shards are similar to the results observed for the quantitative analysis of Fe in the K-411 shards. A comparison of the intensities ratios for the spot and area scans taken at 20 kV and 10 kV are shown in Figs. 12 and 13, respectively, A statistical T test was performed on the data and the results indicate that the means for the 20 kV area and spot analyses are indistinguishable at the 95 % confidence level while the means for 10 kV area and spot analyses are different at the 95 % confidence level with the mean for the spot analyses lower than the mean for the area analyses.

Since subsurface charging effects in the non-conducting glass particles may possibly explain the anomalous Fe data for the glass shards, the Duane Hunt limit was determined from the x-ray continuum for the K-3189 shards analyzed at 10 kV. The values ranged from a low of 9.96 keV to a high of 10.01 keV and do not indicate any significant static charging that would cause the observed anomaly [13].

**Fig. 14:**
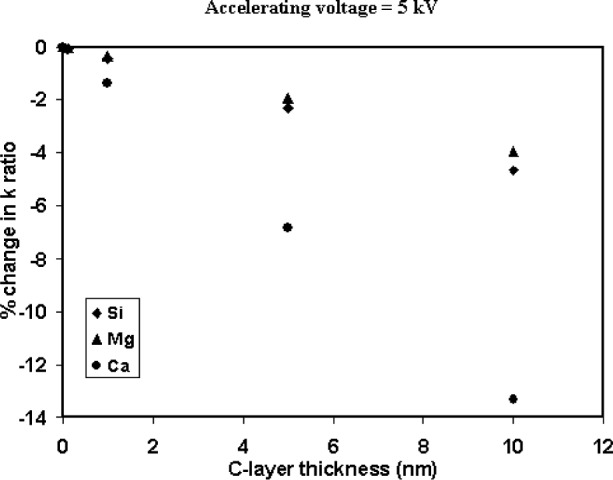
Monte Carlo calculations of the percent change in k-ratios for elements in K-411 glass as a function of carbon thickness.

Finally, the severity of the contamination effect can also be seen in the dramatic reduction of the Ca particle/bulk ratios at 5 kV in Fig. 7. Many of the particle/bulk ratios are less than 0.8 for the smaller particles. In this case, not only is the average beam energy significantly lowered by the contamination but also the overvoltage for Ca Kα x-ray at 5 kV is only 1.24, well below the recommended value. In this instance, the overvoltage is so low that the Ca intensity is extremely sensitive to contamination even in the FEG-SEM with the better chamber vacuum. Monte Carlo calculations for the Ca k-ratio shown in Fig. 14 indicate that a 5 nm C-layer reduces the Ca *k*-ratio by 7 % and a 10 nm layer reduces it by 13 %.

### 1.4 Conclusions

The results of this study indicate that reducing the accelerating voltage of the electron beam for quantitative particle analysis significantly reduces the magnitude of the particle mass and absorption effects. By reducing the beam energy to 10 kV it was possible to quantitatively analyze irregularly-shaped shards of K-411 glass using bulk standards and a conventional ZAF correction procedure. At 10 kV the variation in mass fraction × was only + 0.08 to 0.12 without any corrections for particle size or geometry. This compares to a variation in mass fraction of + 0.26 to 0.60 relative for a similar analysis at 25 kV.

Although the application of low-voltage analysis reduces the effects of particle size and shape it also introduces systematic trends in the analytical results that must be taken into account for accurate quantitative analysis. These trends, which can be significant, are not related to particle effects but are associated with the buildup of contamination under bombardment of the electron beam. The contamination lowers the intensity of the measured x rays by lowering the average energy of the electron beam. This effect increases in magnitude as the accelerating potential of the beam is lowered, being on the order of 5 % for the elements Si and Mg in the K-411 glass microspheres analyzed at 5 kV. The systematic negative errors resulting from contamination are particularly severe for elements that also have low overvoltages such as seen in this study for the Fe data from the shards at 10 kV and the Ca data from the microspheres at 5 kV.

Further reduction of accelerating voltages will only exacerbate the problems associated with contamination buildup on particle surfaces, especially in conventional electron beam instruments where the sample-chamber vacuum is on the order of 10^−4^ Pa to 10^−5^ Pa and on samples where contamination arises from preparation steps. In samples where the particles are inherently “dirty” (for example atmospheric particles with hydrocarbon residues) low-voltage analysis may not be a viable option for quantitative analysis. Contamination will also be a problem for all low-voltage analyses, including bulk materials, in which a high electron dose is used for sample analysis such as a spot beams or small-area rasters.

## Figures and Tables

**Table 1:** Elemental concentrations (mass fraction × 100) for glass K-411

Constituent	Concentration ± 2-sigma
SiO_2_	54.30 ± 0.20
FeO	14.42 ± 0.20
MgO	14.67 ± 0.20
CaO	15.47 ± 0.20

**Table 2:** Elemental glass K-3189 (concentrations in mass fraction × 100)

Constituent	Concentrations
SiO_2_	40.0
Al_2_O_3_	14.0
CaO	14.0
MgO	10.0
TiO_2_	2.0
Fe_2_O_3_	20.0

**Table 3:** Fe/Si and Fe/Ca intensities ratios for glass K-3189 at 20 kV and 10 kV

10kV Fe/Si area	10 kV Fe/Si spot	10 kV Fe/Ca area	10 kV Fe/Ca spot

0.0537 ± 0.0069	0.0443 ± 0.0016	0.221 ± 0.021	0.194 ± 0.0079



20kV Fe/Si area	20 kV Fe/Si spot	20 kV Fe/Ca area	20 kV Fe/Ca spot

0.282 ± 0.034	0.284 ± 0.028	0.650 ± 0.034	0.628 ± 0.037

## Footnotes

1 Certain commercial equipment, instruments, or materials are identified in this paper to foster understanding. Such identification does not imply recommendation or endorsement by the National Institute of Standards and Technology, nor does it imply that the materials or equipment identified are necessarily the best available for the purpose.
